# Epidemiology of Antimicrobial Resistance Genes in *Staphylococcus aureus* Isolates from a Public Database from a One Health Perspective—Sample Origin and Geographical Distribution of Isolates

**DOI:** 10.3390/antibiotics12121654

**Published:** 2023-11-24

**Authors:** Francesca Zaghen, Valerio Massimo Sora, Gabriele Meroni, Giulia Laterza, Piera Anna Martino, Alessio Soggiu, Luigi Bonizzi, Alfonso Zecconi

**Affiliations:** 1 Department of Biomedical, Surgical and Dental Sciences-One Health Unit, School of Medicine, University of Milan, Via Pascal 36, 20133 Milan, Italy; 2 Department of Clinical and Community Sciences, School of Medicine, University of Milan, Via Celoria 22, 20133 Milan, Italy

**Keywords:** *S. aureus*, One Health, antimicrobial resistance, molecular epidemiology, geographical distribution

## Abstract

*Staphylococcus aureus* are commensal bacteria that are found in food, water, and a variety of settings in addition to being present on the skin and mucosae of both humans and animals. They are regarded as a significant pathogen as well, with a high morbidity that can cause a variety of illnesses. The Centers for Disease Control and Prevention (CDC) has listed them among the most virulent and resistant to antibiotics bacterial pathogens, along with *Escherichia coli*, *Staphylococcus*, *Klebsiella pneumoniae*, *Acinetobacter baumannii*, *Pseudomonas aeruginosa*, *Enterococcus faecalis*, and *Enterococcus faecium*. Additionally, *S. aureus* is a part of the global threat posed by the existence of antimicrobial resistance (AMR). Using 26,430 *S. aureus* isolates from a global public database (NPDIB; NCBI Pathogen Detection Isolate Browser), epidemiological research was conducted. The results corroborate the evidence of notable variations in isolate distribution and ARG (Antimicrobial Resistance Gene) clusters between isolate sources and geographic origins. Furthermore, a link between the isolates from human and animal populations is suggested by the ARG cluster patterns. This result and the widespread dissemination of the pathogens among animal and human populations highlight how crucial it is to learn more about the epidemiology of these antibiotic-resistance-related infections using a One Health approach.

## 1. Introduction

*Staphylococcus aureus* is a commensal bacterium present on the skin and mucosae in both humans and animals, but it can also be found in food, water, and various other environments [1,2]. Unfortunately, it is also a major pathogen of great morbidity, leading to a wide range of infections including bacteremia, infective endocarditis, complicated skin and soft tissue infections, pleuropulmonary infections, urinary tract infections, toxic shock syndrome, and prosthetic device infections [3]. Most cases of infection are observed in healthcare and community settings [4] and it has been estimated that the global mortality due to *S. aureus* infections reached approximately 1 million in 2019 [5].

*S. aureus* is also present in almost every animal species, from wild animals [6] to livestock and pets [7,8], and it can lead to different kinds of infections that can be a health and economic burden, especially in farm animals, including mastitis in ruminants [9], septicemia, osteoarticular infections, and pododermatitis in poultry [10,11,12,13], exudative epidermitis in piglets [14], and cutaneous abscesses [15] and mastitis in rabbits [16]. 

*S. aureus*, with its enterotoxins, is also considered one of the principal pathogens responsible for foodborne diseases [17]. Indeed, 241,000 illnesses per year are estimated in the United States [18], while in Europe, the officially diagnosed cases in 2021 were 640 with more than 50 hospitalizations [19]; these latter small numbers probably underestimate the frequency of the infections due to their mild symptoms not requiring medical attention, thus leading to unreported foodborne infections in many countries.

*S. aureus* is included in the list of most virulent and antimicrobic resistant bacterial pathogens (ESKAPE) by the Centers for Disease Control and Prevention (CDC), and *S. aureus* is part of the worldwide threat represented by AMR [1,19]. This latter problem and the large diffusion of these pathogens among human and animal populations support the importance of gaining information on the epidemiology of these infections using a One Health approach. This approach is fundamental to developing efficient control strategies that consider isolates from both humans and animals, as well as the possible risks related to bacteria and ARGs spreading via the environment. The access to publicly available databases, collecting isolates from numerous locations and sources, may help to investigate the distribution of AMR genes among isolates, as shown in a previous study [20]. This paper is focused on reporting the results of the epidemiological analysis on the distribution of *S. aureus* AMR genes based on the isolation characteristics of geography, source, and clinical characteristics.

## 2. Results

### 2.1. Data Description

**Figure 1:**
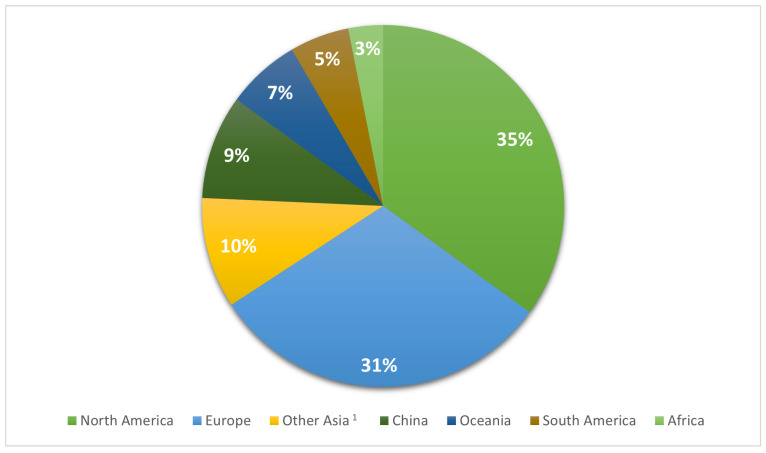
Distribution of *S. aureus* isolates by geographical region. ^1^ Other Asia category includes countries with a frequency <100: Saudi Arabia, Bangladesh, Cambodia, United Arab Emirates, Jordan, Hong Kong, Kazakhstan, Kuwait, Lebanon, Nepal, Oman, Pakistan, Russia, Singapore, Syria, Sri Lanka, South Korea, Thailand, Taiwan, Turkey, and Vietnam.

We considered the worldwide public database NCBI Pathogen Detection Isolate Browser (NPDIB). On the 30 April 2022, the public database included 35,026 *S. aureus* isolates. The isolates were classified into three groups: human-associated (HUA), non-human-associated (NHA), and unknown (UNK). The HUA category contains samples taken in healthcare settings, while the NHA category includes isolates from animals, food, and the environment. Isolates with little or no information about the origin of isolation were labeled as unknown origin, thus forming the UNK category. Isolates without information on their geographical origin were not considered in the analysis. After data verification, we included 26,430 isolates in the epidemiological analysis. Most of the isolates submitted were from North America (USA, Canada, and Mexico) and Europe with 35% and 30.8% of the isolates, respectively. Asia accounted for about 20% of the isolates, whereas Oceania, South America, and Africa had the lowest percentages, with frequencies of 3–7% (Figure 1). 

Table 1 reports the distribution of the isolates among the different geographical regions by the source characteristics: 63% of them were classified as HUA, 7.8% were NHA, while the remaining isolates had an unknown origin. The HUA group was the most frequent in each geographical area; a significant statistical difference among NHA, HUA, and UNK isolates using the χ^2^ test (α = 0.05) was observed in each area, except Africa.

### 2.2. European Isolates

**Figure 2:**
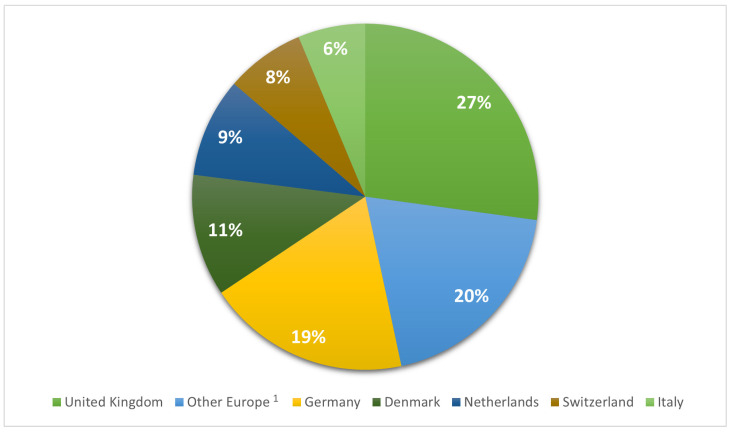
Distribution of *S. aureus* isolates in Europe. ^1^ Other Europe category includes countries with a frequency <100: Austria, Belgium, Belarus, Croatia, Finland, Greece, Latvia, Lithuania, Luxembourg, Poland, Portugal, Czech Republic, Romania, Serbia, Slovenia, and Hungary.

Given the substantial number of isolates originating from Europe and the United States, we comprehensively analyzed this dataset. In Europe (Figure 2), the UK had 2212 (27%), followed by Germany with 1547 (19%), Denmark with 928 (11.4%), the Netherlands with 754 (9.3%), Switzerland with 599 (7.4%), and Italy with 515 (6.3%). Other European states had lower isolate frequencies. The proportion of UK isolates was significantly larger than the other countries, but when the ratio cases/population was considered, the proportion of cases was higher for Denmark (154 records/million people), Switzerland (68 records/million people) and the Netherlands (42 records/million people), while in the UK, 33 records/million people were registered and in Germany, the number was 19 records/million people.

The trend observed in the worldwide distribution among NHA, HUA, and UNK isolates was observed for the European data as well (Table 2), where a significant statistical difference between the three groups was present in every area considered. 

### 2.3. USA Isolates

**Figure 3:**
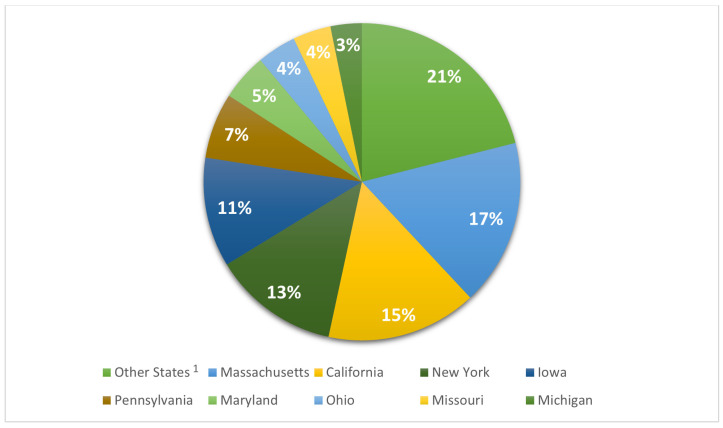
Distribution of *S. aureus* isolates in USA. ^1^ Other States category includes: Alabama, Alaska, Arizona, Arkansas, Colorado, Connecticut, Delaware, Florida, Georgia, Hawaii, Idaho, Illinois, Indiana, Kansas, Kentucky, Louisiana, Maine, Minnesota, Mississippi, Montana, Nebraska, Nevada, New Hampshire, New Jersey, New Mexico, North Carolina, North Dakota, Oklahoma, Oregon, Rhode Island, South Carolina, South Dakota, Tennessee, Texas, Utah, Vermont, Virginia, Washington, West Virginia, Wisconsin, and Wyoming.

Within the different states of the USA, Massachusetts had the highest frequency of isolates (1530, 17%), followed by California with 15% of the US isolates (1388 isolates), New York state with 13% (1160 isolates), and Iowa with 11% (1007 isolates). The remaining states had frequencies lower than 10% (Figure 3). For the statistical analysis, all US states with a frequency <2% were included in the category “Other States”.

Table 3 reports the distribution and the statistical differences observed among NHA, HUA, and UNK isolates among the states, which had similar results compared to Europe. The statistical analysis results showed that a statistically significant difference among the three groups of isolates was observed for each state, except for New York, Iowa, Pennsylvania, and Missouri. 

In addition, 314 records/million people were submitted from Iowa, a typical agricultural state, while Massachusetts, which supplied the highest number of isolates, had a proportion of 218 records/million people, while New York had 59 records/million people and California had 35 records/million people. 

### 2.4. Resistance Gene Distribution

Among the 67 ARGs reported in the database, only those with a total prevalence >2% were considered for the statistical analyses. Furthermore, regulatory genes such as *blaI*, *blaR1* for *blaZ*, and *mecI* and *mecR1* for *mecA* were excluded from this study. The most frequent ARGs identified were those conferring resistance to the tetracycline antimicrobial family with more than 77,000 positive identifications and those responsible for resistance against penams and fosfonic acid, with 51,459 and 45,659 identifications, respectively; ARGs related to resistance to aminoglycosides and fluoroquinolones both had more than 36,000 positive identifications, and genes related to resistance to macrolides had 15,784 positive identifications. Our prior study examined single ARG prevalence in depth [20]. 

### 2.5. Cluster Analyses

**Figure 4:**
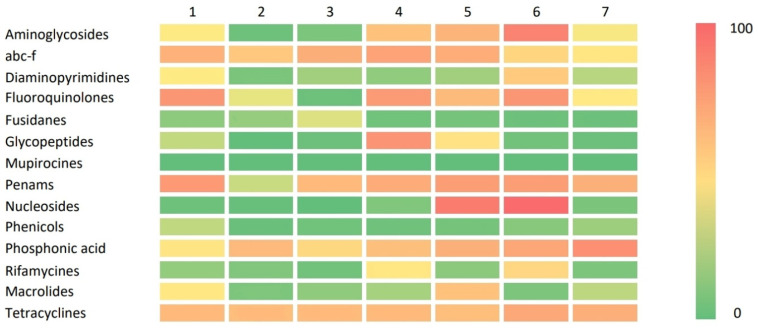
Graphical representation (heat map) of the antibiotic classes’ related ARG frequencies in each cluster [20].

**Figure 5:**
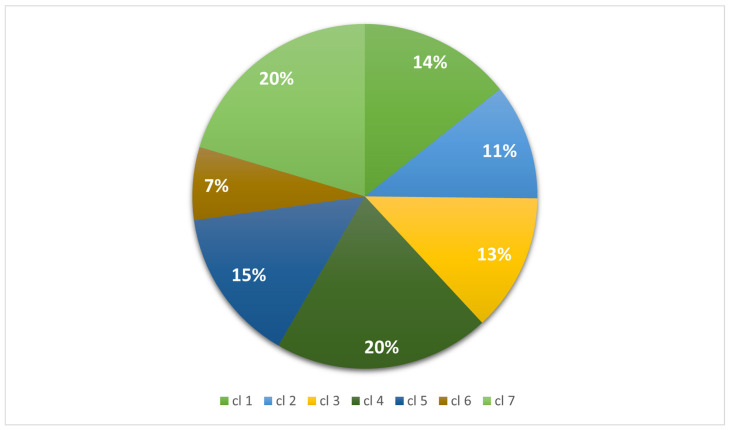
Cluster distribution related to the current database. The cluster analysis process was reported in the companion paper [20].

In order to identify a possible pattern in the distribution of the ARGs, a cluster analysis was performed on the dataset, not only to recognize a particular asset in the AMR of the various isolates, but also to identify potential associations between the different patterns and the source or the region of origin of the isolates. The analysis identified seven different clusters based on the presence of the ARGs described in Supplementary Table S1. In Figure 4, we graphically represent the ARG rates divided according to the relative antibiotic class and clusters to visually describe the ARG distribution among all seven clusters, as previously reported [20]. Briefly, Clusters 2 and 3 were identified as the clusters with the lowest presence of ARGs, while Clusters 4, 5, and 6 had high rates of ARGs related to nine different antibiotic classes. Clusters 1 and 7 showed a mild resistance pattern with a progressive increase in ARG frequency from Cluster 7 to Cluster 1. In Figure 5, the distribution of the seven clusters identified in the subset of isolates considered in this paper are visualized. 

#### 2.5.1. Association between Gene Cluster and Geographical Area of Submission 

The statistical analysis was performed to identify possible associations between the origin of the isolates and the cluster membership (Table 4, Table 5 and Table 6). The statistical analysis showed large and significant variations among clusters in relation to the geographical origin (Table 4). Indeed, North America had the highest significant prevalence of Clusters 4 and 5 (64.9% and 73.5%, respectively); Cluster 1 was more prevalent in Europe, with 2034 isolates, representing more than half of all isolates in this cluster (53.7%); Cluster 2 with 1153 isolates (40.3%), Cluster 7 with 2228 isolates (41.4%), and Cluster 3 with 1138 isolates (33.3%) were also more frequently reported in Europe, even if with lower prevalence. Cluster 6 showed the highest prevalence in Other Asian Countries with 1019 isolates (56.3%). 

#### 2.5.2. European and USA Isolates

**Figure 6:**
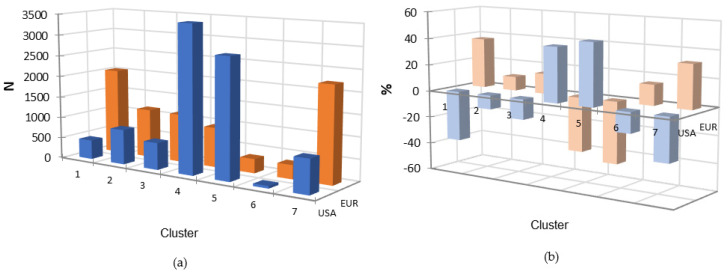
Comparison between the distribution of clusters (**a**) and residues (difference between expected and observed frequency) (**b**) among European (orange colored bars) and USA (blue colored bars) *S. aureus* isolates.

Since most of the isolates were reported from Europe and the USA, and the numbers between these two areas were comparable, we also analyzed the different distribution of clusters between these two areas. The results of the χ^2^ analysis (Figure 6a) and of the residues (Figure 6b) confirmed significant differences in the cluster frequencies between the European and USA isolates. These differences are particularly significant for Clusters 1, 5, 6, and 7.

When the cluster distribution was analyzed within European countries, great differences were also observed (Table 5). Indeed, Cluster 1 was mainly recovered in the UK, while Germany supplied about one third of the isolates of Cluster 7, as well as Switzerland for Cluster 4. More generally, each country appeared to be characterized by one or two clusters with a prevalence largely higher than all of the others. 

The same analysis applied to the USA (Table 6) gave similar results, with the distribution of clusters largely associated with a specific state. Indeed, Cluster 4 was mainly associated with Massachusetts isolates, Cluster 5 with New York isolates, Cluster 6 with California, and Cluster 7 with Iowa isolates.

### 2.6. Isolates from Humans (Clinical Sources)

We also investigated human (clinical) isolates in detail, which are those with more precise characterization in the database. They were more frequently classified in Clusters 4, 5, and 7 (Table 7), and the statistical analysis of the frequencies among geographical areas supports the difference observed in the general database. Most isolates in North America were classified in Clusters 4 and 5, while Cluster 1 was the most frequent in Europe, supplying nearly 50% of the isolates classified in this cluster. Cluster 2 isolates came mainly from North America, Europe, and Asian countries. 

When the statistical analysis was performed within European countries, the results showed that 75% of the isolates in Cluster 1 were from the UK. Clusters 3 and 7 were more frequently associated with Germany, while Italy supplied about one third of the Cluster 6 isolates (Table 8). The same analysis performed on the USA isolates (Table 9) showed that Clusters 4 and 5 represented more than 70% of the total HUA isolates, Massachusetts was the area where Cluster 4 isolates were more frequently isolated, while New York State was the major source of Cluster 5 isolates. 

### 2.7. Isolates from Animals, Food, and Environment 

As stated before, the amount of NHA isolate was very low, representing only 7.8% of the whole database. Nonetheless, the data reported in Supplementary Table S2 show that Cluster 7 is mainly associated with China and Europe, with 42.2% and 31.2% of the isolates, respectively, while Cluster 2 is prevalent in Europe and North America (40.7% and 36.1%, respectively). Notably, the distribution of the NHA isolates in Europe is characterized by the complete absence of them in Clusters 5 and 6 (Supplementary Table S3), while Cluster 7 is more present in Germany (61.3%). In the USA, most of the isolates fall in Cluster 5 (166) with a great contribution from Maryland (42.2%), while there are no isolates in Cluster 6 (Supplementary Table S4). 

## 3. Discussion

*S. aureus* is a highly adapted microorganism, with different lineages associated with specific hosts [21]; while a change in the major host is rare, spillover events can be more common and lead to infection in unusual hosts [22]. The risk of transmission should consider not only zoonotic or anthropozoonotic (reverse zoonotic) infections, but also the ARG spread among species, through pathways that still need to be investigated [23]. The presence of these risks supports the importance of epidemiological studies on the characteristics and distribution of the isolates with different genetic patterns [24]. Publicly available datasets, collecting isolates from various countries and sources, allow the monitoring of the epidemiology of *S. aureus* and can help foresee changes in the distribution of different lineages and ARGs, as already observed for other pathogens, like *S. agalactiae* [25]. 

The analysis of the database considered in this study supports the evidence of significant differences in the distribution of isolates and ARG clusters among geographical areas of origin and sources of the isolates. Geographical differences in the genetic characteristics of isolates were already known in the case of bovine mastitis [26,27,28], but these differences were only recently investigated in the case of human isolates [24,29,30]. More than 60% of the records originated in Europe and the USA, suggesting the relevance of the problem of AMR spread in these areas, but the proportion of records from Asia (19%) is not negligible and confirms the increasing importance of *S. aureus* infections and AMR spread in this area as well [25,31,32,33]. 

As reported in other studies, there is a scarcity of information derived from low- to middle-income countries, also evident in this study, reflecting the limits of the local healthcare systems where resources for the control and prevention of AMR are limited [34,35]. Indeed, one of the limits of this study is represented by the voluntariness of the submissions of the isolates, and the uneven frequencies of reporting information among countries could be attributed to economic limitations, missed diagnoses, or the lack of interest in sharing the data.

Most records are related to human clinical cases, and relatively few to environmental, animal, or food isolates. This imbalance could be a source of bias in the analysis when the different sources are compared; the close values of the NHA isolates from North America, the USA, and China suggest that an imbalance between NHA and HUA isolates is common in these highly populated areas. This may also be due to the low prevalence of severe illnesses in humans, usually not requiring hospitalization, leading to an underestimation of the frequency of these infections. 

Despite the population size and public health conditions being similar within different European countries, the frequency and relevance of the problem seem to be different, suggesting the presence of local factors that could influence the spread and characteristics of the infections. For example, the Netherlands and Denmark have significant food animal populations, mainly cows and pigs, that may play a role in the epidemiology of *S. aureus* infections, as already shown for MRSA infections [36]. 

These results support the importance of a One Health approach to investigate these infections and the need for a larger number of isolates from animal, environmental, and food sources to confirm the pattern that emerged from the data considered in this study. The analysis of the distribution of ARG clusters among and within continents fully supports the previous observations and suggests that the circulation of the different isolates is associated with relatively small areas, and the development of AMR may be mainly due to the therapeutical protocols applied locally and cannot be generalized. Indeed, the results of this study suggest that the ARG clusters characterized by higher AMR (Clusters 4, 5, and 6) [20] are recovered with significantly higher frequency in North America, while the other clusters, characterized by lower AMR patterns, originate mainly from Europe. These differences are also supported by the evidence of different distributions of the clusters even when relatively homogeneous economic and political areas (USA and Europe) were compared. 

In Europe, the prevalent cluster among HUA isolates is Cluster 1; this cluster is mainly associated with ARGs that are resistant to fluoroquinolones, penams, and tetracycline, while other prevalent clusters are numbers 2 and 7 that involve resistance toward fosfonic acid, tetracycline, and penams. It is interesting to note that in European states, the NHA isolates with ARGs are also included in Clusters 2 and 7, which are characterized by quite a high prevalence of ARGs resistant to rifamycin and fosfonic acid, which are not allowed for veterinary use [37]. Clusters 5 and 6 are both characterized by ARGs for nucleosides [20]: in Europe, this antimicrobial class is not authorized for veterinary use, and this could explain why we did not find any NHA isolates in Clusters 5 and 6 among the European isolates [37].

Similarly, in the USA, Massachusetts was the major source of Cluster 4, while New York State was a major source for Cluster 5. These differences may result from a higher transmission frequency of genetically similar isolates in the specific geographical area and/or from applying different therapeutical protocols among the different states. Indeed, Cluster 4 is characterized by a high frequency of genes leading to fluoroquinolone and glycopeptide resistance, while Cluster 5 is characterized by a high frequency of genes related to penam and nucleoside resistance [20]. It is important to highlight that Iowa is the single state with the highest frequency of insolates in Cluster 7, supporting the hypothesis of an association with livestock. Indeed, this cluster is related mostly to ARGs directed against tetracycline and penams [20], which are largely applied in livestock treatments [38].

Cluster 7 was also frequently observed within Asian isolates, suggesting a similar epidemiological pattern to the other continents. 

Overall, the results of this study support the evidence that the occurrence of isolates with peculiar characteristics, including higher morbidity and AMR, may be identified, and should be considered [29]. It also implies that the preventive measures to reduce the occurrence and development of AMR should be aimed at the clusters of *S. aureus* with the highest frequency at the local level [24].

## 4. Materials and Methods

### 4.1. NCBI Pathogen Detection Isolate Browser and Antibacterial Data (NPDIB)

More than one million isolates from 80 different bacteria are available from the NCBI pathogen detection isolate browser (NPDIB). The strains submitted to the database, used in this epidemiological study, were analyzed using the same parameters described in a previous study [20]. Identification data from the database were exported and organized into columns with Microsoft Excel™. Each column represented an AMR gene, and the value of the cell was associated with a dichotomic variable: 1 if the ARG was present, and 0 if it was not. The information in the other columns (e.g., source of isolation, geographical area) were kept as in the original database.

### 4.2. Statistical Analysis

The statistical analysis was performed on SPSS 28.0.1.1 (IBM Corp., Armonk, NY, USA, 2022). We used a χ^2^ test with Bonferroni adjustment in order to analyze the frequency distribution. When the cell numerosity was below 6, a Fisher’s exact test was applied instead of a χ^2^ test. With the aim of classifying isolates based on the different combinations of AMR genes, cluster analysis was performed using the following parameters: squared Euclidean distance, Ward’s agglomeration method, and truncation at 20% of total distance [39]. Cluster analysis is a multivariate technique that allows the grouping of isolates based on their characteristics (e.g., AMR genes).

## 5. Conclusions

The availability of public databases that collect genetic information on pathogens allows the epidemiology of different pathogens and the ARG patterns to be investigated. The data analysis of *S. aureus* isolates from NPDIB databases confirmed that both isolate distribution and ARGs patterns are specific to different geographical areas. This can be related to the different diagnostic capabilities, local therapeutical approaches, and availability of antimicrobial molecules, as well as to the epidemiology of these bacteria in animals, particularly livestock. From a One Health point of view, this type of analysis is crucial, because it allows similarities and differences to be identified among human, animal, and environmental isolates, as well as the possible interactions between the groups.

The quantity and quality of information collected in the database should be implemented in order to intensify the surveillance of *S. aureus* resistance, not only in the human medicine purview, but also in veterinary medicine and in the field of food and environmental contamination. A larger number of isolates from different sources and a periodical analysis of prevalence and ARGs patterns are pivotal in developing effective control programs.

## Figures and Tables

**Table 1:** Geographical distribution and statistical differences of non-human-associated, human-associated, and unknown isolates.

Geographical Region		NHA ^1^	HUA	UNK	Total
North America	*n* %	595 ^a,2^ (6.4)	7940 ^b^ (77.7)	1470 ^c^ (15.9)	9255 (100)
Europe	*n* %	490 ^a^ (6)	3441 ^b^ (42.3)	4213 ^c^ (51.7)	8144 (100)
Other Asia ^3^	*n* %	135 ^a^ (5.2)	1832 ^b^ (70.3)	639 ^c^ (24.5)	2606 (100)
China	*n* %	685 ^a^ (27.8)	802 ^b^ (32.6)	974 ^c^ (39.6)	2461 (100)
Oceania	*n* %	85 ^a^ (4.9)	1472 ^b^ (84.6)	183 ^c^ (10.5)	1740 (100)
South America	*n* %	13 ^a^ (0.9)	1369 ^b^ (98.2)	12 ^c^ (0.9)	1394 (100)
Africa	*n* %	56 ^a^ (6.7)	545 ^a^ (65.7)	229 ^a^ (27.6)	830 (100)

^1^ NHA = non-human-associated, HUA = human-associated, UNK = unknown origin. ^2^ Values with different letter superscripts among lines statistically differ at χ^2^ test (α = 0.05). ^3^ Other Asia category includes Saudi Arabia, Bangladesh, Cambodia, United Arab Emirates, Jordan, Hong Kong, Kazakhstan, Kuwait, Lebanon, Nepal, Oman, Pakistan, Russia, Singapore, Syria, Sri Lanka, South Korea, Thailand, Taiwan, Turkey, and Vietnam.

**Table 2:** European distribution of NHA, HUA, and UNK isolates.

State		NHA ^1^	HUA	UNK	Total
United Kingdom	*n* %	73 ^a,2^ (3.3)	1440 ^b^ (65.1)	699 ^a^ (31.6)	2212 (100)
Other Europe ^3^	*n* %	129 ^a^ (8.1)	593 ^b^ (37.3)	867 ^c^ (54.6)	1589 (100)
Germany	*n* %	135 ^a^ (8.7)	586 ^b^ (37.9)	826 ^c^ (53.4)	1547 (100)
Denmark	*n* %	3 ^a^ (0.3)	153 ^b^ (16.5)	722 ^c^ (83.2)	928 (100)
Netherlands	*n* %	8 ^a^ (1.1)	282 ^b^ (37.4)	464 ^c^ (61.5)	754 (100)
Switzerland	*n* %	81 ^a^ (13.5)	115 ^b^ (19.2)	403 ^c^ (67.3)	599 (100)
Italy	*n* %	61 ^a^ (11.8)	272 ^b^ (52.8)	182 ^c^ (35.3)	515 (100)

^1^ NHA = non-human-associated, HUA = human-associated, UNK = unknown origin. ^2^ Values with different letter superscripts among lines statistically differ at χ^2^ test or Fisher’s exact test (α = 0.05). ^3^ Other Europe category includes Austria, Belgium, Belarus, Croatia, Finland, Greece, Latvia, Lithuania, Luxembourg, Poland, Portugal, Czech Republic, Romania, Serbia, Slovenia, and Hungary.

**Table 3:** USA distribution of NHA, HUA, and UNK isolates.

State		NHA ^1^	HUA	UNK	Total
Other States ^2^	*n* %	243 ^a,3^ (12.8)	1153 ^b^ (60.7)	503 ^c^ (26.5)	1899 (100)
Massachusetts	*n* %	0 ^a^ (0.0)	1239 ^b^ (81)	291 ^c^ (19)	1530 (100)
California	*n* %	0 ^a^ (0.0)	829 ^b^ (59.7)	559 ^c^ (40.3)	1388 (100)
New York	*n* %	74 ^a^ (6.4)	1068 ^a^ (92.1)	18 ^b^ (1.6)	1160 (100)
Iowa	*n* %	0 ^a^ (0.0)	1003 ^b^ (99.6)	4 ^a^ (0.4)	1007 (100)
Pennsylvania	*n* %	2 ^a^ (0.3)	593 ^b^ (98.5)	7 ^a^ (1.2)	602 (100)
Maryland	*n* %	121 ^a^ (28.2)	282 ^b^ (65.7)	26 ^c^ (6.1)	429 (100)
Ohio	*n* %	0 ^a^ (0.0)	344 ^b^ (94.8)	19 ^c^ (5.2)	363 (100)
Missouri	*n* %	0 ^a^ (0.0)	352 ^b^ (100)	0 ^a^ (0.0)	352 (100)
Michigan	*n* %	3 ^a^ (1.1)	270 ^b^ (95.1)	11 ^a^ (3.9)	284 (100)

^1^ NHA = non-human-associated, HUA = human-associated, UNK = unknown origin. ^2^ Other States category includes Alabama, Alaska, Arizona, Arkansas, Colorado, Connecticut, Delaware, Florida, Georgia, Hawaii, Idaho, Illinois, Indiana, Kansas, Kentucky, Louisiana, Maine, Minnesota, Mississippi, Montana, Nebraska, Nevada, New Hampshire, New Jersey, New Mexico, North Carolina, North Dakota, Oklahoma, Oregon, Rhode Island, South Carolina, South Dakota, Tennessee, Texas, Utah, Vermont, Virginia, Washington, West Virginia, Wisconsin, and Wyoming. ^3^ Values with different letter superscripts among lines statistically differ at χ^2^ test or Fisher’s exact test (α = 0.05).

**Table 4:** Geographical distribution of clusters and statistical difference among geographical regions within each cluster.

Geographical Region	Cluster 1 *n* (%)	Cluster 2 *n* (%)	Cluster 3 *n* (%)	Cluster 4 *n* (%)	Cluster 5 *n* (%)	Cluster 6 *n* (%)	Cluster 7 *n* (%)
North America	456 ^e,1^ (12.1)	977 ^c^ (34.1)	651 ^c^ (19.1)	3471 ^f^ (64.9)	2802 ^f^ (73.5)	59 ^f^ (3.3)	839 ^e^ (15.6)
Europe	2034 ^b^ (53.7)	1153 ^a^ (40.3)	1138 ^d^ (33.3)	933 ^a^ (17.5)	331 ^d^ (8.7)	327 ^d^ (18.1)	2228 ^a^ (41.4)
Other Asia ^2^	229 ^a^ (6.0)	193 ^b^ (6.7)	225 ^c^ (6.6)	236 ^b,d^ (4.4)	255 ^a^ (6.7)	1019 ^c^ (56.2)	449 ^d^ (8.3)
China	622 ^b^ (16.4)	159 ^b^ (5.5)	468 ^b^ (13.7)	359 ^e^ (6.7)	28 ^b^ (0.7)	38 ^b^ (2.1)	787 ^b^ (14.6)
Oceania	335 ^c^ (8.8)	122 ^b^ (4.3)	401 ^a^ (11.7)	131 ^c,d^ (2.4)	43 ^e^ (1.1)	33 ^b^ (1.8)	675 ^c^ (12.5)
South America	38 ^a^ (1.0)	127 ^b,c^ (4.4)	344 ^c^ (10.1)	136 ^a,b,c,d^ (2.5)	289 ^e^ (7.6)	256 ^e^ (14.2)	204 ^d^ (3.8)
Africa	75 ^a^ (2.0)	136 ^a^ (4.7)	188 ^a,b^ (5.5)	84 ^a,b,c,d^ (1.6)	66 ^a^ (1.7)	77 ^a^ (4.3)	204 ^a^ (3.8)
Total	3789 (100)	2867 (100)	3415 (100)	5350 (100)	3814 (100)	1809 (100)	5386 (100)

^1^ Values with different letter superscripts among rows statistically differ at χ^2^ test or Fisher’s exact test (α = 0.05). ^2^ Other Asia category includes Saudi Arabia, Bangladesh, Cambodia, United Arab Emirates, Jordan, Hong Kong, Kazakhstan, Kuwait, Lebanon, Nepal, Oman, Pakistan, Russia, Singapore, Syria, Sri Lanka, South Korea, Thailand, Taiwan, Turkey, and Vietnam.

**Table 5:** Geographical distribution of clusters in Europe and statistical differences among countries.

State	Cluster 1 *n* (%)	Cluster 2 *n* (%)	Cluster 3 *n* (%)	Cluster 4 *n* (%)	Cluster 5 *n* (%)	Cluster 6 *n* (%)	Cluster 7 *n* (%)
United Kingdom	1243 ^e,1^ (61.1)	377 ^b^ (32.6)	150 ^d^ (13.2)	273 ^e^ (29.3)	11 ^b^ (3.3)	53 ^b^ (16.2)	105 ^f^ (4.7)
Other Europe ^2^	408 ^d^ (20.1)	252 ^a^ (21.9)	266 ^c^ (23.3)	157 ^c,e^ (16.8)	89 ^a^ (26.9)	107 ^d^ (32.8)	310 ^d^ (13.9)
Germany	227 ^b^ (11.2)	164 ^b^ (14.2)	210 ^b,c^ (18.5)	90 ^b^ (9.6)	90 ^a^ (27.2)	57 ^b^ (17.4)	709 ^b^ (31.8)
Denmark	90 ^a^ (4.4)	153 ^a^ (13.3)	289 ^a^ (25.4)	12 ^a^ (1.3)	61 ^a^ (18.4)	3 ^a^ (0.9)	320 ^a^ (14.4)
Netherlands	7 ^c^ (0.3)	82 ^b^ (7.1)	102 ^b,c^ (9)	0 ^d^ (0.0)	1 ^b^ (0.3)	0 ^a^ (0.0)	562 ^c^ (25.2)
Switzerland	11 ^c^ (0.5)	56 ^b^ (4.9)	56 ^b,d^ (4.9)	364 ^f^ (39)	41 ^a^ (12.4)	23 ^b,d^ (7)	48 ^e^ (2.2)
Italy	48 ^a^ (2.4)	69 ^a,b^ (6)	65 ^b,c^ (5.7)	37 ^b,c^ (4)	38 ^a^ (11.5)	84 ^c^ (25.7)	174 ^a^ (7.8)
Total	2034 (100)	1153 (100)	1138 (100)	933 (100)	331 (100)	327 (100)	2228 (100)

^1^ Values with different letter superscripts among rows statistically differ at χ^2^ test or Fisher’s exact test (α = 0.05). ^2^ Other Europe category includes Austria, Belgium, Belarus, Croatia, Finland, Greece, Latvia, Lithuania, Luxembourg, Poland, Portugal, Czech Republic, Romania, Serbia, Slovenia, and Hungary.

**Table 6:** Geographical distribution of clusters in USA and statistical differences among states.

State	Cluster 1 *n* (%)	Cluster 2 *n* (%)	Cluster 3 *n* (%)	Cluster 4 *n* (%)	Cluster 5 *n* (%)	Cluster 6 *n* (%)	Cluster 7 *n* (%)
Other States ^1^	95 ^b,d,f,h,2^ (20.9)	290 ^a^ (35.2)	253 ^d^ (39.9)	496 ^c^ (14.4)	387 ^b^ (13.8)	13 ^a,b,c^ (22.7)	365 ^b^ (46.1)
Massachusetts	15 ^i^ (3.3)	7 ^g^ (0.8)	18 ^a^ (2.8)	1191 ^b^ (34.4)	268 ^b^ (9.6)	1 ^c^ (1.8)	30 ^a,b,c^ (3.8)
California	87 ^a,b,c,d,e,f,g^ (19.1)	170 ^a,b^ (20.6)	10 ^a^ (1.6)	587 ^a^ (17)	492 ^a^ (17.5)	20 ^a^ (35.1)	22 ^a^ (2.8)
New York	101 ^c,g^ (22.2)	122 ^a,b,c^ (14.8)	97 ^b^ (15.3)	114 ^e^ (3.3)	661 ^c^ (23.6)	13 ^a,b^ (22.7)	52 ^c^ (6.6)
Iowa	31 ^h^ (6.8)	90 ^b,c,d,e,f^ (10.9)	78 ^b^ (12.3)	402 ^a^ (11.6)	227 ^b^ (8.1)	1 ^b,c^ (1.8)	178 ^b^ (22.5)
Pennsylvania	54 ^a,c,e,g^ (11.9)	37 ^c,d,e,f^ (4.5)	36 ^b^ (5.7)	231 ^a^ (6.7)	221 ^a^ (7.9)	5 ^a,b,c^ (8.8)	18 ^a,c^ (2.3)
Maryland	35 ^e,f,g^ (7.7)	18 ^e,f^ (2.2)	21 ^b^ (3.3)	85 ^c^ (2.5)	251 ^c^ (9)	3 ^a,b,c^ (5.3)	16 ^a,c^ (2)
Ohio	11 ^b,d,f,h,i^ (2.4)	32 ^a,b,c,d,e,f^ (3.9)	19 ^b^ (3)	143 ^a^ (4.1)	122 ^a^ (4.4)	0 ^a,b,c^ (0.0)	36 ^d^ (4.5)
Missouri	9 ^d,h,i^ (2)	46 ^a,b^ (5.6)	86 ^c^ (13.6)	20 ^e^ (0.6)	120 ^a^ (4.3)	1 ^a,b,c^ (1.8)	70 ^b^ (8.8)
Michigan	17 ^a,b,c,d,e,f,g,h^ (3.7)	12 ^d,f^ (1.5)	16 ^b^ (2.5)	185 ^d^ (5.4)	49 ^b^ (1.8)	0 ^a,b,c^ (0.0)	5 ^a,c^ (0.6)
Total	455 (100)	824 (100)	634 (100)	3454 (100)	2798 (100)	57 (100)	792 (100)

^1^ Other States category includes Alabama, Alaska, Arizona, Arkansas, Colorado, Connecticut, Delaware, Florida, Georgia, Hawaii, Idaho, Illinois, Indiana, Kansas, Kentucky, Louisiana, Maine, Minnesota, Mississippi, Montana, Nebraska, Nevada, New Hampshire, New Jersey, New Mexico, North Carolina, North Dakota, Oklahoma, Oregon, Rhode Island, South Carolina, South Dakota, Tennessee, Texas, Utah, Vermont, Virginia, Washington, West Virginia, Wisconsin, and Wyoming. ^2^ Values with different letter superscripts among rows statistically differ at χ^2^ test or Fisher’s exact test (α = 0.05).

**Table 7:** Distribution and statistical differences of HUA isolates among clusters in geographical regions.

Geographical Region	Cluster 1 *n* (%)	Cluster 2 *n* (%)	Cluster 3 *n* (%)	Cluster 4 *n* (%)	Cluster 5 *n* (%)	Cluster 6 *n* (%)	Cluster 7 *n* (%)
North America	344 ^a,1^ (14.7)	631 ^a^ (39.6)	508 ^d^ (25.5)	2824 ^d^ (76)	2222 ^e^ (76.7)	49 ^b^ (3.3)	612 ^d^ (23.3)
Europe	1165 ^f^ (49.6)	601 ^d^ (37.7)	481 ^b^ (24.3)	331 ^a^ (8.9)	157 ^c^ (5.4)	95 ^d^ (6.4)	611 ^c^ (23.3)
Other Asia ^2^	161 ^c^ (6.8)	43 ^c^ (2.7)	37 ^c^ (1.9)	187 ^a,c^ (5)	141 ^a^ (4.9)	989 ^e^ (66.7)	274 ^c^ (10.4)
China	328 ^d^ (13.9)	56 ^a,b^ (3.5)	89 ^b^ (4.5)	109 ^c^ (2.9)	5 ^b^ (0.2)	4 ^b,c^ (0.3)	211 ^a^ (8)
Oceania	288 ^e^ (12.2)	83 ^b^ (5.2)	386 ^a^ (19.5)	87 ^b^ (2.4)	42 ^c^ (1.4)	28 ^c,d^ (1.9)	558 ^b^ (21.2)
South America	37 ^b^ (1.6)	121 ^a^ (7.6)	340 ^a^ (17.1)	133 ^a,c^ (3.6)	287 ^d^ (9.9)	250 ^f^ (16.9)	201 ^c^ (7.7)
Africa	29 ^a,b,c,d^ (1.2)	59 ^a^ (3.7)	142 ^a^ (7.2)	45 ^a,b,c^ (1.2)	43 ^a^ (1.5)	67 ^a^ (4.5)	160 ^a^ (6.1)
Total	2352 (100)	1594 (100)	1983 (100)	3716 (100)	2897 (100)	1482 (100)	2627 (100)

^1^ Values with different letter superscripts among rows statistically differ at χ^2^ test or Fisher’s exact test (α = 0.05). ^2^ Other Asia category includes Saudi Arabia, Bangladesh, Cambodia, United Arab Emirates, Jordan, Hong Kong, Kazakhstan, Kuwait, Lebanon, Nepal, Oman, Pakistan, Russia, Singapore, Syria, Sri Lanka, South Korea, Thailand, Taiwan, Turkey, and Vietnam.

**Table 8:** Distribution and statistical differences of HUA isolates among clusters in European countries.

State	Cluster 1 *n* (%)	Cluster 2 *n* (%)	Cluster 3 *n* (%)	Cluster 4 *n* (%)	Cluster 5 *n* (%)	Cluster 6 *n* (%)	Cluster 7 *n* (%)
United Kingdom	880 ^e,1^ (75.5)	244 ^b^ (40.6)	39 ^f^ (8.1)	217 ^g^ (65.7)	7 ^c^ (4.5)	25 ^a,c^ (26.3)	28 ^c^ (4.6)
Other Europe ^2^	122 ^d^ (10.5)	122 ^b,c^ (20.3)	97 ^a,c,d,e^ (20.2)	69 ^f,g^ (20.8)	21 ^a^ (13.4)	14 ^a,b,c^ (14.7)	148 ^a^ (24.2)
Germany	74 ^b^ (6.4)	106 ^b,c^ (17.6)	132 ^d,e^ (27.4)	15 ^d,e^ (4.5)	47 ^b^ (29.9)	14 ^a,b,c^ (14.7)	198 ^b^ (32.4)
Denmark	53 ^a^ (4.5)	9 ^a^ (1.5)	37 ^a,b,c,d,e^ (7.7)	2 ^a,b,c,d,e^ (0.6)	14 ^a^ (8.9)	2 ^a,b,c^ (2.1)	36 ^a,b^ (5.9)
Netherlands	7 ^c^ (0.6)	72 ^c^ (12)	97 ^b^ (20.2)	0 ^b,e^ (0.0)	0 ^c^ (0.0)	0 ^c^ (0.0)	106 ^b^ (17.3)
Switzerland	5 ^b,c^ (0.4)	6 ^a^ (1)	31 ^a,b,c,d,e^ (6.4)	8 ^a,c,d,f,g^ (2.4)	41 ^d^ (26.1)	7 ^b,d^ (7.4)	17 ^a^ (2.8)
Italy	24 ^b^ (2.1)	42 ^a,b,c^ (7)	48 ^c,e^ (10)	20 ^c,f^ (6)	27 ^b^ (17.2)	33 ^d^ (34.8)	78 ^a,b^ (12.8)
Total	1165 (100)	601 (100)	481 (100)	331 (100)	157 (100)	95 (100)	611 (100)

^1^ Values with different letter superscripts among rows statistically differ at χ^2^ test or Fisher’s exact test (α = 0.05). ^2^ Other Europe category includes Austria, Belgium, Belarus, Croatia, Finland, Greece, Latvia, Lithuania, Luxembourg, Poland, Portugal, Czech Republic, Romania, Serbia, Slovenia, and Hungary.

**Table 9:** Distribution and statistical differences of HUA isolates among clusters in USA.

State	Cluster 1 *n* (%)	Cluster 2 *n* (%)	Cluster 3 *n* (%)	Cluster 4 *n* (%)	Cluster 5 *n* (%)	Cluster 6 *n* (%)	Cluster 7 *n* (%)
Other States ^1^	56 ^b,d,f,g,h,i,j,k,2^ (16.4)	166 ^a^ (27)	145 ^d^ (29.3)	363 ^f^ (12.9)	204 ^b,c^ (9.2.)	10 ^a,b,c^ (20.8)	209 ^b^ (34.7)
Massachusetts	10 ^l^ (2.9)	6 ^g^ (1)	13 ^b^ (2.6)	974 ^b^ (34.7)	207 ^c^ (9.3)	1 ^c^ (2.1)	28 ^a^ (4.7)
California	49 ^a,b,c,d,e,f,g,h,i,j,k^ (14.3)	113 ^a,b^ (18.4)	9 ^a,b^ (1.8)	340 ^a^ (12.1)	292 ^a^ (13.2)	14 ^a^ (29.1)	12 ^a^ (2)
New York	96 ^c^ (28.1)	107 ^a,b,c,e^ (17.4)	85 ^c^ (17.1)	112 ^e^ (4)	616 ^d^ (27.7)	13 ^a,b^ (27.1)	39 ^a^ (6.5)
Iowa	31 ^h,i,j,k^ (9)	89 ^b,c,d,e,f^ (14.5)	78 ^c^ (15.7)	401 ^a^ (14.3)	227 ^b^ (10.2)	1 ^b,c^ (2.1)	176 ^b^ (29.3)
Pennsylvania	54 ^a,c,e^ (15.7)	36 ^c,d,e,f^ (5.9)	35 ^c^ (7)	226 ^a,f^ (8)	219 ^a^ (9.9)	5 ^a,b,c^ (10.4)	18 ^a^ (3)
Maryland	22 ^a,b,c,d,e,f,g^ (6.4)	13 ^f^ (2.1)	11 ^a,c^ (2.2)	56 ^c^ (2)	166 ^d^ (7.5)	3 ^a,b,c^ (6.3)	11 ^a,c^ (1.8)
Ohio	8 ^b,d,f,g,h,i,j,k,l^ (2.3)	28 ^a,b,c,d,e,f^ (4.6)	19 ^c^ (3.8)	136 ^a,f^ (4.8)	119 ^a^ (5.4)	0 ^a,b,c^ (0.0)	34 ^c^ (5.7)
Missouri	9 ^d,g,i,k,l^ (2.6)	46 ^a,b^ (7.5)	86 ^e^ (17.3)	20 ^e^ (0.7)	120 ^a^ (5.4)	1 ^a,b,c^ (2.1)	70 ^b^ (11.6)
Michigan	8 ^e,f,g,j,k,l^ (2.3)	10 ^d,f^ (1.6)	16 ^c,d^ (3.2)	183 ^d^ (6.5)	49 ^b,c^ (2.2)	0 ^a,b,c^ (0.0)	4 ^a^ (0.7)
Total	343 (100)	614 (100)	497 (100)	2811 (100)	2219 (100)	48 (100)	601 (100)

^1^ Other States category includes Alabama, Alaska, Arizona, Arkansas, Colorado, Connecticut, Delaware, Florida, Georgia, Hawaii, Idaho, Illinois, Indiana, Kansas, Kentucky, Louisiana, Maine, Minnesota, Mississippi, Montana, Nebraska, Nevada, New Hampshire, New Jersey, New Mexico, North Carolina, North Dakota, Oklahoma, Oregon, Rhode Island, South Carolina, South Dakota, Tennessee, Texas, Utah, Vermont, Virginia, Washington, West Virginia, Wisconsin, and Wyoming. ^2^ Values with different letter superscripts among rows statistically differ at χ^2^ test or Fisher’s exact test (α = 0.05).

## Data Availability

Publicly available datasets were analyzed in this study. The data can be found here: https://www.ncbi.nlm.nih.gov/pathogens/isolates/#taxgroup_name:%22Staphylococcus%20aureus%22 (accessed on 30 April 2022) and https://worldpopulationreview.com/ (accessed on 30 April 2022).

## Supplementary Materials

The following supporting information can be downloaded at https://www.mdpi.com/article/10.3390/antibiotics12121654/s1: Supplementary Table S1. ARG frequencies according to the antibiotic class and cluster; *n* = number of positive isolates. The genes with the highest frequency for each antibiotic class are represented in bold type. Supplementary Table S2. Distribution and statistical difference of NHA isolates among clusters in different geographical regions. Supplementary Table S3. Distribution and statistical difference of NHA isolates among European countries. Supplementary Table S4. Distribution and statistical difference of NHA isolates among clusters in USA.
