# Supplementary material for: Epidemiology of Antimicrobial Resistance Genes in Staphylococcus aureus Isolates from a Public Database from a One Health Perspective—Sample Origin and Geographical Distribution of Isolates
Source: Antibiotics (Basel). 2023 Nov 24;12(12):1654. doi: 10.3390/antibiotics12121654 (PMC10740469; doi:10.3390/antibiotics12121654)
Supplement: Supplementary file 1 [file antibiotics-12-01654-s001.zip › antibiotics-2686668-supplementary.pdf]

Supplementary Table S1. ARG frequencies according to the antibiotic class and cluster; *n* = number of positive isolates. The genes with the highest frequency for each antibiotic class are represented in bold types [20].

Antibiotic Class	Gene	Cluster						
	ARG (*n*)	1	2	3	4	5	6	7
aminoglycosides	***ant(9)-la* (9179)**	**0.3%**	**0.3%**	**0.1%**	**95.7%**	**20%**	**94.7%**	**9.4%**
	ant(6)-la (7310)	10.6%	0.1%	0%	0.3%	94.3%	99.2%	8.7%
	aadD1 (7033)	17.7%	1.1%	3.3%	70.5%	19.9%	23.6%	5.3%
	aph(3′)-lla (6984)	0.7%	0.5%	0.2%	2.9%	96.6%	99.3%	7.9%
	aac(6′)-le/aph(2″)-la (6102)	18.3%	1.7%	4%	23.3%	10.6%	92.1%	14.3%
antibiotics targeting protein synthesis	***Abc-f* (13816)**	**49.7%**	**34.2%**	**52.1%**	**59.3%**	**53.2%**	**24.3%**	**13.3%**
diaminopyrimidines	***dfrG* (3657)**	**16.4%**	**2.2%**	**6.2%**	**0.8%**	**6.9%**	**64.2%**	**9%**
	dfrS1 (681)	2.7%	0.6%	1.4%	4.9%	0.7%	1.1%	1.3%
fluoroquinolones	***parC* (17446)**	**97.7%**	**13.3%**	**1.4%**	**92.2%**	**64.6%**	**96.0%**	**18.7%**
	gyrA (16601)	95.0%	10.3%	0.2%	91.4%	61.1%	95.8%	14.6%
	parE (2126)	15.2%	0.8%	0.3%	13.5%	3.7%	14.3%	0.3%
fusidanes	***fusC* (807)**	**2.7%**	**3.1%**	**7.6%**	**0.9%**	**1.2%**	**0.7%**	**0.7%**
glycopeptides	***bleO* (5197)**	**5.9%**	**0.1%**	**0.6%**	**70.1%**	**16.2%**	**1%**	**0.6%**
mupirocines	***mup(A)* (1421)**	**5%**	**1.1%**	**1.5%**	**4.0%**	**7.0%**	**23.1%**	**0.4%**
	ileS (1000)	1.6%	0.4%	0.1%	11.9%	1.5%	4.9%	0.4%
nucleosides	***sat4* (5986)**	**0.7%**	**0.3%**	**0%**	**1.8%**	**87.2%**	**98.7%**	**1.4%**
penams	***mecA* (22968)**	**94.4%**	**18.2%**	**32.0%**	**93.3%**	**91.3%**	**98.2%**	**53.5%**
	blaZ (22850)	96.1%	0.4%	99.4%	56.5%	97.6%	27.1%	60.1%
	blaPC1 (5641)	7.9%	0.1%	0.2%	10.7%	1.7%	66.2%	39.3%
phenicols	***fex(A)* (806)**	**10%**	**0.4%**	**0.8%**	**0%**	**0%**	**0%**	**2.6%**
	catA (795)	1.5%	0.4%	0.9%	1.6%	2.3%	4.6%	4.6%
phosphonic acid	***fos(B)* (21257)**	**33.1%**	**51%**	**64.2%**	**96.9%**	**93.3%**	**99.5%**	**34.1%**
	murA (15048)	10.3%	48.4%	5.5%	16.1%	57.6%	73.3%	93.8%
	glpT (9354)	0.3%	34.3%	1.7%	8%	2%	0.3%	92.6%
rifamycines	***rpoB* (1819)**	**3.1%**	**1.8%**	**0.9%**	**13.5%**	**2.5%**	**25.1%**	**1.6%**
macrolides	*mph(C)* (4401)	4.2%	0.6%	0.6%	6.2%	87.7%	1.9%	0.3%
	msr(A) (4571)	4.4%	0.9%	1.5%	6.4%	87.8%	2.1%	1.3%
	erm(A) (112)	0.3%	0.2%	0.1%	95.6%	19.9%	95.2%	8.5%
	***erm(C)* (5685)**	**50.7%**	**4.8%**	**10.5%**	**7.8%**	**11.4%**	**3.3%**	**15.7%**
	erm(B) (1015)	2.5%	0.9%	1%	0%	0.2%	0%	9.8%
tetracyclines	*tet (38)* (33893)	99.6%	99.9%	100%	99.6%	93.9%	98.5%	100%
	tet(k) (4869)	8.5%	3.4%	9.1%	3.5%	8.4%	40.2%	30.7%
	tet (L) (1087)	9.9%	0.3%	0.7%	0.3%	2.6%	0.1%	4.7%
	tet(m) (3621)	1.1%	1.6%	1.6%	11.9%	1%	43.9%	22.3%
	***mepA* (34205)**	**99.9%**	**100%**	**99.9%**	**100%**	**100%**	**100%**	**99.8%**

Supplementary Table S2. Distribution and statistical differences of NHA isolates among clusters in different geographical region.

Geographical Region	Cluster 1 *n* (%)	Cluster 2 *n* (%)	Cluster 3 *n* (%)	Cluster 4 *n* (%)	Cluster 5 *n* (%)	Cluster 6 *n* (%)	Cluster 7 *n* (%)
North America	15$^{c, d}$[1] (8.7)	190$^{a, c}$ (36.1)	88$^{a, d}$ (21.4)	46^{a} (69.6)	166^{f} (86.9)	0^{b} (0.0)	90^{a} (13.1)
Europe	8^{c} (4.7)	214^{b} (40.7)	49$^{c, d}$ (11.9)	5^{b} (7.6)	0$^{b, e}$ (0.0)	0$^{a, b}$ (0.0)	214^{b} (31.2)
Other Asia[2]	10$^{a, d}$ (5.8)	48$^{a, b, c}$ (9.1)	54^{b} (13.1)	0^{b} (0.0)	11^{c} (5.8)	0$^{a, b}$ (0.0)	12^{a} (1.7)
China	131^{b} (76.1)	38^{d} (7.2)	201^{b} (48.8)	10^{b} (15.2)	12$^{d, e}$ (6.3)	3$^{a, b}$ (75)	290^{b} (42.2)
Oceania	0$^{c, d}$ (0.)	16^{c} (3)	1^{c} (0.2)	0$^{a, b}$ (0.0)	0$^{a, b, c, d, e}$ (0.0)	0$^{a, b}$ (0.0)	68^{c} (9.9)
South America	0$^{a, b, c, d}$ (0.0)	5$^{a, b, c}$ (0.9)	4$^{a, b, d}$ (1)	0$^{a, b}$ (0.0)	1$^{a, c, d, f}$ (0.5)	0$^{a, b}$ (0.0)	3$^{a, b}$ (0.4)
Africa	8$^{a, b}$ (4.7)	16^{c} (3.0)	15$^{a, b}$ (3.6)	5^{a} (7.6)	1$^{a, b, c, d, e}$ (0.5)	1^{a} (25)	10^{a} (1.5)
Total	172 (100)	527 (100)	412 (100)	66 (100)	191 (100)	4 (100)	687 (100)

[1] Values with different letter superscripts among lines statistically differ at χ2 test or Fisher's exact test (α=0.05).

[2] Other Asia includes: Saudi Arabia, Bangladesh, Cambodia, United Arab Emirates, Jordan, Hong Kong, Kazakhstan, Kuwait, Lebanon, Nepal, Oman, Pakistan, Russia, Singapore, Syria, Sri Lanka, South Korea, Thailand, Taiwan, Turkey and Viet Nam.

Supplementary Table S3. Distribution and statistical differences of NHA isolates among European countries.

State	Cluster 1 *n* (%)	Cluster 2 *n* (%)	Cluster 3 *n* (%)	Cluster 4 *n* (%)	Cluster 5 *n* (%)	Cluster 6 *n* (%)	Cluster 7 *n* (%)
United Kingdom	1^{a}[1] (12.5)	52^{a} (24.4)	17^{a} (34.7)	0^{a} (0.0)	-	-	3^{d} (1.4)
Other Europe[2]	3^{a} (37.5)	82^{a} (38.3)	16^{a} (32.7)	4^{a} (80)	-	-	24$^{a, d}$ (11.2)
Germany	4^{a} (50)	0^{c} (0.0)	0^{b} (0.0)	0^{a} (0.0)	-	-	131^{c} (61.3)
Denmark	0^{a} (0.0)	1$^{a, b}$ (0.5)	0$^{a, b}$ (0.0)	0^{a} (0.0)	-	-	2$^{a, b, c}$ (0.9)
Netherlands	0^{a} (0.0)	8^{a} (3.7)	0$^{a, b}$ (0.0)	0^{a} (0.0)	-	-	0$^{a, b, d}$ (0.0)
Switzerland	0^{a} (0.0)	48$^{a, b}$ (22.4)	5$^{a, b}$ (10.2)	1^{a} (20)	-	-	27$^{a, b}$ (12.6)
Italy	0^{a} (0.0)	23^{b} (10.7)	11^{a} (22.4)	0^{a} (0.0)	-	-	27^{b} (12.6)
Total	8 (100)	214 (100)	49 (100)	5 (100)	-	-	214 (100)

[1] Values with different letter superscripts among rows statistically differ at χ2 test or Fisher's exact test (α = 0.05).

[2] Other Europe includes: Austria, Belgium, Belarus, Croatia, Finland, Greece, Latvia, Lithuania, Luxembourg, Poland, Portugal, Czech Republic, Romania, Serbia, Slovenia and Hungary.

Supplementary Table S4. Distribution and statistical differences of NHA isolates among clusters in USA countries.

State	Cluster 1 *n* (%)	Cluster 2 *n* (%)	Cluster 3 *n* (%)	Cluster 4 *n* (%)	Cluster 5 *n* (%)	Cluster 6 *n* (%)	Cluster 7 *n* (%)
Other States[1]	0[b 2] (0.0)	41[b, c] (73.2)	67[b] (79.8)	17[b, c] (38.6)	53[b] (31.9)	-	65[b] (83.4)
New York	4[a] (26,7)	8[a, c] (14.3)	9[a, b] (10.7)	1[c] (2.3)	43[a] (25.9)	-	9[a, b] (11.5)
Pennsylvania	0[a] (0.0)	1[b, c] (1.8)	0[a, b] (0.0)	1[a, b] (2.3)	0[a, b] (0.0)	-	0[a, b] (0.0)
Maryland	11[a] (73.3)	4[a] (7.1)	8[a] (9.5)	25[a] (56.8)	70[a] (42.2)	-	3[a] (3.8)
Michigan	0[a, b] (0.0)	2[b] (3.6)	0[a, b] (0.0)	0[a, b, c] (0.0)	0[a, b] (0.0)	-	1[b] (1.3)
Total	15 (100)	56 (100)	84 (100)	44 (100)	166 (100)	-	78 (100)

[1] Other states includes: Alabama, Alaska, Arizona, Arkansas, Colorado, Connecticut, Delaware, Florida, Georgia, Hawaii, Idaho, Illinois, Indiana, Kansas, Kentucky, Louisiana, Maine, Minnesota, Mississippi, Montana, Nebraska, Nevada, New Hampshire, New Jersey, New Mexico, North Carolina, North Dakota, Oklahoma, Oregon, Rhode Island, South Carolina, South Dakota, Tennessee, Texas, Utah, Vermont, Virginia, Washington, West Virginia, Wisconsin, Wyoming.

[2] Values with different letter superscripts among lines statistically differ at χ^2 test or Fisher's exact test (α = 0.05).
